# Assessing the educational quality of YouTube videos on celiac plexus blocks: Expert review and AI-based evaluation^☆^

**DOI:** 10.1016/j.inpm.2026.100740

**Published:** 2026-01-30

**Authors:** Julia Zhu, David Hao, Robert Jason Yong

**Affiliations:** a Harvard Medical School, USA; b Department of Anesthesiology, Mass General Brigham, Boston, MA, USA

**Keywords:** Celiac plexus block, YouTube, Medical education, Online health information

## Abstract

**Background:**

YouTube is an open-access platform increasingly used for both medical and patient education, but its user-generated content is not subject to peer review and shows wide variability in accuracy and quality. Celiac plexus blocks are technically complex procedures that are presented on YouTube, yet the educational quality of these instructional videos has not yet been systematically evaluated.

**Objective:**

To evaluate the educational quality of YouTube videos on celiac plexus blocks and to explore the utility of ChatGPT-4o as a secondary, adjunctive tool for assessing video quality.

**Methods:**

YouTube was searched on June 2nd, 2025 using the keywords “celiac plexus neurolysis,” “celiac block for cancer pain,” “celiac plexus block,” and “celiac plexus injection.” The 17 most-viewed videos were independently evaluated by two board-certified chronic pain physicians and by ChatGPT-4o using a modified DISCERN scale (mDISCERN), the Global Quality Scale (GQS), and a usefulness classification.

**Results:**

Based on human expert ratings, only 18 % of videos contained highly reliable information as assessed by the mDISCERN scale, and 24 % demonstrated moderate to excellent information quality on the Global Quality Scale. Overall, 65 % of videos were classified as useful. Inter-rater reliability between human experts ranged from poor to moderate across the three scales of evaluation, while agreement between human expert and ChatGPT-4o assessment was poor.

**Conclusions:**

The educational quality of YouTube videos on celiac plexus blocks was generally poor. Unlike similar studies investigating other procedures, the quality of videos produced by physician and hospital sources was not better than that of videos by nonacademic sources. These findings highlight the need to improve the quality of educational content produced by physicians, hospitals, and professional societies.

## 1 Introduction

With 2.54 billion users and 44.9 % of all global internet users visiting the site on a monthly basis, YouTube is currently the third-most-used social media platform [1]. It serves as a hub of open-access, user-generated videos, emerging as a tool for information sharing beyond entertainment. Patients, clinicians, and students alike often use YouTube for sharing their experiences, medical learning, and disseminating medical information [[2], [3], [4]]. A prior study found that an estimated 40 % of providers encourage their patients to research disease processes on social media [5]. Moreover, the popularity of video formatted medical education has been growing, and surveys of medical students reveal that many consider YouTube a major learning resource [3,6,7].

Despite its accessibility, YouTube content is not subject to peer review, raising concerns about the educational quality and accuracy of its medical videos. In the digital era, health-related misinformation can spread rapidly through social media platforms [[8], [9], [10]]. A growing body of literature has evaluated the instructional value of YouTube videos across various medical specialties. Most studies have found that these videos are often incomplete or inaccurate, with higher-quality content more likely to originate from academic or institutional sources [[11], [12], [13], [14], [15], [16]]. Notably, increased user engagement does not reliably reflect educational quality. Rather, view counts are more strongly associated with how long the video has been available on the platform [11,17].

Artificial intelligence (AI) has concurrently played a larger role in medical education in recent years [18]. Large language models (LLMs) have been increasingly used for both content generation and information gathering [19]. Survey data shows that ChatGPT, in particular, has been utilized by medical students for summarizing information, generating differential diagnoses, and conducting literature reviews [20,21]. Early research further suggests that LLMs may approximate expert judgement but can be highly variable, depending on the model, version, and prompt [[22], [23], [24], [25], [26], [27]]. Their accessibility and scalability already position them as potentially valuable proxies for screening and triaging large volumes of user-generated content, such as YouTube videos, for medical learning. These models, however, carry inherent limitations: potential hallucinations, biases, and variable alignment with current evidence-based guidelines limited by their training data cutoffs.

The broad trends identified in the quality of medical learning YouTube videos similarly hold true for regional anesthesia techniques and topics, including erector spinae plane blocks, epidural anesthesia, and brachial plexus blocks [11,12,[28], [29], [30]]. However, no literature has specifically investigated the quality of videos discussing celiac plexus blocks, an intervention with many techniques, including posterior para-aortic, anterior para-aortic, posterior transaortic, trans-intervertebral disc, and endoscopic approaches, and additional variance in imaging guidance and drug dosing [31]. These procedural differences were found to reflect individual preference, institutional practice, and experience with the technique [[32], [33], [34]].

Given the variety of techniques used to perform celiac plexus blocks, the educational quality and accuracy of instructional content are particularly important on widely accessible platforms like YouTube. In this study, we evaluated the quality of the most-viewed YouTube videos on celiac plexus blocks and examined the relationship between video source, educational quality, and user engagement. We additionally examined AI-based assessment of quality. Together, these findings may inform efforts to improve online pain procedure educational content and clarify the potential role of AI in content evaluation.

## 2 Methods

We performed a cross-sectional analysis of YouTube videos related to celiac plexus blocks. On June 2nd, 2025, YouTube was searched using the terms: “celiac plexus neurolysis,” “celiac block for cancer pain,” “celiac plexus block,” and “celiac plexus injection.” Videos were eligible for inclusion if their primary focus was celiac plexus block or neurolysis. Exclusion criteria included non-English language, absence of verbal audio, duplicate videos, or classification as YouTube Shorts. Using the platform's view-count filter, the 20 most-viewed videos meeting inclusion criteria were initially selected. Three videos representing distinct postings with significant duplicate content were subsequently excluded, resulting in a final sample of 17 videos.

For each video, the following data was recorded: video length, total views, likes, dislikes, number of channel subscribers, time since upload (in months), and upload source. Upload sources were categorized as physician/hospital, media organization, or “other” when the source did not fit pre-defined categories or could not be determined. Specifically, a media organization was defined as a source independent of a specific academic affiliation with a primary focus of producing media content (e.g., news company, podcast group, etc.).

Video quality was assessed using three tools: the modified DISCERN scale (mDISCERN), the Global Quality Scale (GQS), and a usefulness classification. Each video was independently evaluated by two board-certified chronic pain physicians (D.H. and R.J.Y.) and by an AI model. Inter-rater reliability between the human reviewers was calculated to assess scoring consistency, and discrepancies were resolved through discussion to achieve consensus. For AI-based evaluation, ChatGPT-4o was provided with a table containing video links, video characteristics, and detailed descriptions of the mDISCERN and GQS scoring criteria. The model was then prompted to generate mDISCERN and GQS scores for each video (for the full prompt, see Appendix).

The mDiscern scale is a validated 5-item scale widely used to assess the quality of online content [35]. It was adapted from the original 16-item DISCERN scale, developed by Charnock et al. to evaluate written health information [36]. The scale has five criteria: 1) the aims are clear and achieved; 2) reliable sources of information are used; 3) the information presented is balanced and unbiased; 4) additional sources of information are listed for patient reference; and 5) areas of uncertainty are mentioned. Each criterion met receives 1 point, totaling a score ranging from 0 to 5. A score of 3 or higher generally indicates that the content is highly reliable [35].

The GQS is a 5-point Likert scale developed by Bernard et al. to evaluate the overall quality and accessibility of online resources for patients. Each rating is defined as follows: 1) very poor quality (poor flow, missing information, and not at all useful for patients); 2) poor quality (poor flow, some information, and little usefulness for patients); 3) moderate quality (moderate flow, some important information adequately explained, and somewhat useful for patients); 4) good quality (good flow, most information present, and useful for patients); and 5) excellent quality (excellent flow with information very useful for patients) [37].

Videos were also categorized using a usefulness classification. A video was deemed useful if it contained at least one accurate statement regarding the mechanism, indications, adverse effects, or outcomes of celiac plexus blocks. Videos were classified as misleading if they contained at least one inaccurate or exaggerated claim, and as neither useful nor misleading if no substantive medical information was provided.

Statistical analyses were performed using the Statistical Package for the Social Sciences (SPSS) version 30.0.0.0 (IBMCorp, Armonk, NY, USA). Descriptive statistics were used to summarize video characteristics. Continuous variables were reported as medians with interquartile ranges (IQR), and categorical variables were presented as frequencies and percentages. The Mann-Whitney *U* test was used to compare video characteristics across rating categories, and Pearson correlation coefficients were calculated to assess relationship between video characteristics, with statistical significance defined as *P* < 0.05.

Inter-rater reliability between human reviewers was assessed using Cohen's kappa (κ) for categorical assessments (usefulness classification) and intraclass correlation coefficients (ICCs) for ordinal measures (mDISCERN and GQS). Specifically, the ICC was calculated using a two-way mixed model with absolute agreement. Agreement was further assessed between human consensus ratings and AI-generated scores. ICC or κ values greater than 0.75 were considered excellent agreement, values between 0.40 and 0.75 moderate, and values below 0.40 poor.

Institutional Review Board approval was not required for this study, as it involved analysis of publicly available YouTube videos and did not include human subjects.

## 3 Results

Video characteristics, including duration on YouTube, video length, number of views, number of likes, and subscriber count, along with human expert ratings are summarized in Table 1. Among the 17 videos evaluated, 18 % (n = 3) were rated as highly reliable, defined as an mDISCERN score of 3 or more (score distribution: 5, n = 0; 4, n = 2; 3, n = 1; 2, n = 7; 1, n = 7; 0, n = 0). For information quality, 24 % (n = 4) of the videos were rated as having at least moderate quality, defined as a GQS score of 3 of more (score distribution: 5, n = 0; 4, n = 0; 3, n = 4; 2, n = 10; 1, n = 3).

**Table 1:** YouTube video characteristics and quality.

	Median (IQR)
Duration on YouTube (months)	133 (57.5–154.5)
Video length (seconds)	284 (183.5–569)
Number of views	9903 (5138.5–26795.5)
Numbers of likes	38 (12.5–137)
Number of dislikes	0 (0–0)
Number of subscribers	3500 (242–8150)
mDISCERN score (human-rated consensus)	2 (1–2)
GQS score (human-rated consensus)	2 (2–2.5)

Using the usefulness classification, 65 % (n = 11) of the videos were deemed useful. The remaining videos were classified as misleading (n = 3) or neither useful nor misleading (n = 3).

AI-based evaluation found no videos to be highly reliable (mDISCERN score of ≥3). Most videos received low reliability scores (score distribution: 5, n = 0; 4, n = 0; 3, n = 0; 2, n = 5; 1, n = 11; 0, n = 1). For information quality, AI rated 47 % (n = 8) of videos as having at least moderate quality (GQS ≥3), including two videos with a GQS score of 4 and six with a score of 3. The other videos were rated as poor (GQS score of 2, n = 7) or very poor (GQS score of 1, n = 2).

Physician or hospital sources accounted for 10 of the 17 videos, while four were created by media organizations. Of the physician/hospital videos, one was rated as highly reliable by human reviewers (mDISCERN score ≥3), two demonstrated at least moderate quality (GQS score ≥3), and seven were considered useful. In contrast, the AI model did not rate any physician/hospital videos as highly reliable (mDISCERN ≥3) but assigned a GQS of 3 or higher to five of these videos. Among the four videos produced by media organizations, human reviewers rated one video as highly reliable, three as having at least moderate quality, and two as useful. AI-based evaluation identified no media organization videos as highly reliable and rated one as having at least moderate quality.

No statistically significant differences were observed in general video characteristics, including duration on YouTube, video length, number of views, number of likes, and subscriber count, when videos were stratified by expert-rated educational quality using mDISCERN (≥3 versus <3), GQS (≥3 versus <3), or usefulness classification (useful versus misleading or neither). For AI-based ratings, the only significant association was that videos with a GQS score greater than or equal to 3 were shorter than those with lower GQS scores (*P* = 0.007). There was no significant relationship between total view count and time since upload on YouTube (*P* = 0.468).

Inter-rater reliability for the usefulness classification demonstrated poor agreement (Cohen's kappa = 0.325). Agreement between human reviewers was moderate for mDISCERN scoring (single-measures ICC = 0.617) and poor for GQS scoring (single-measures ICC = 0.302). Agreement between the human consensus ratings and AI assessments was poor for both mDISCERN (ICC = 0.111) and GQS (ICC = 0.051) scoring.

## 4 Discussion

Prior studies across multiple medical domains have consistently demonstrated that YouTube-based educational content is often of suboptimal quality [[11], [12], [13], [14], [15]]. Consistent with this literature, we found that the educational quality of YouTube videos addressing celiac plexus blocks was generally poor. Using mDISCERN, GQS, and a usefulness classification, only 18 % of videos were rated as highly reliable and just 24 % demonstrated at least moderate information quality. These findings suggest that many videos lack appropriate sourcing, balance, or acknowledgment of uncertainty. Improving the inclusion of references to reliable sources represents a clear opportunity to enhance content quality.

Similar to prior studies, traditional indicators of user engagement such as likes, views, and subscriber count were not associated with higher educational quality. While this may indicate limited ability of viewers to accurately judge content quality, it likely also reflects the complex and opaque YouTube recommendation algorithm. As a result, engagement metrics should not be interpreted as indicators of reliability.

Unlike previous studies, the quality of videos published by physician/hospital sources were not consistently higher than those produced by media organizations or other sources. This complicates recommendations that viewers rely on source type as a proxy for reliability. Given their expertise, physicians and hospitals arguably bear a greater responsibility to ensure the accuracy of publicly available educational content. A straightforward, actionable step toward improvement is the consistent inclusion of cited sources and references.

Inter-rater reliability between human reviewers ranged from poor to moderate agreement across evaluation tools, underscoring variability in expert judgment even among subspecialists. This variability suggests that assessment of procedural education quality may be inherently subjective. The relatively coarse granularity of existing assessment instruments may further limit their ability to capture nuanced elements of procedural teaching.

Agreement between AI-based assessment tools and human consensus ratings was poor for both the mDISCERN and GQS assessment tools. This discrepancy highlights current limitations of generative AI models like ChatGPT in performing critical evaluations. Unlike prior models, ChatGPT-4o is capable of interpreting audiovisual input [38]. However, in this study, the model was provided only with video URLs and extracted metadata. Because ChatGPT cannot “open” and watch videos in the traditional sense, its evaluations are likely more reflective of user input or metadata encoded by YouTube.

While a previous study evaluating tonsillectomy-related YouTube video content found better agreement between human experts and ChatGPT-4, their model was prompted with a full video transcript [39]. This difference underscores the importance of prompt design and input selection for optimizing large language model evaluative performance. Of note, the only significant finding from the Mann-Whitney U tests was that videos with higher GQS scores were shorter in length, suggesting that AI-derived ratings were not solely driven by metadata or engagement-related inputs.

This study has several limitations. The relatively small sample size may limit generalizability. However, our focus on the most-viewed videos aligns with prior methodology and reflects the practical influence of YouTube's recommendation algorithm [28,30]. Moreover, videos beyond the top-viewed group demonstrated a marked decline in view count, further supporting this approach. Compared with more common procedures such as epidurals or brachial plexus injections, celiac plexus blocks appear to occupy a more niche space, with lower overall viewership and engagement [11,30].

Additionally, despite their use in prior, methodologically similar studies, mDISCERN and GQS are validated tools solely for evaluating the quality of general health information [26,29,30,[35], [36], [37]]. They have not been validated for assessing procedural or technical instruction. The three scoring methods used in this study also did not take into consideration video audiovisuals (e.g., image quality or procedural sequencing), which can impact successful information delivery and viewer engagement. Lastly, by applying uniform evaluation criteria to all potential viewer groups — patients, trainees, and clinicians — without tailoring the assessment to the specific informational needs or baseline knowledge of each subgroup, we implicitly assumed a homogeneous audience. Collectively, these constraints may limit our ability to fully and accurately assess a video's educational quality.

## 5 Conclusion

This study demonstrates that the educational quality of the most-viewed YouTube videos on celiac plexus blocks is generally poor, with low information reliability and inconsistent quality despite moderate rates of perceived usefulness. Traditional engagement metrics and video sources were not reliable indicators of educational quality, underscoring the difficulty viewers face with distinguishing accurate content from incomplete or misleading material. Furthermore, agreement between expert reviewers was variable, highlighting the inherent subjectivity and limitations of existing evaluation instruments. AI-based assessments also showed poor alignment with expert judgement, emphasizing that current LLMs are not reliable substitutes for expert appraisal.

As open-access video platforms and AI-assisted tools increasingly shape medical learning and content, our findings underscore the continued importance of critical appraisal by consumers of medical content. In this context, AI-based tools alone are not sufficient for reliably assessing information quality related to complex procedural education. Accordingly, physicians, professional societies, and hospitals should prioritize improving the rigor, transparency, and sourcing of publicly available educational content, with the goal of ensuring that content originating from these sources is more consistently aligned with established measures of reliability and educational quality.

## Disclosure/funding

Robert Jason Yong reports a relationship with Medtronic Inc. that includes: consulting or advisory.

Robert Jason Yong reports a relationship with Nevro that includes: consulting or advisory.

Robert Jason Yong reports a relationship with Abbott that includes: consulting or advisory.

Robert Jason Yong reports a relationship with Boston Scientific that includes: consulting or advisory.

Robert Jason Yong reports a relationship with Biotronik that includes: consulting or advisory.

## Appendix

The following prompt was used with ChatGPT-4o:

“I would like to have you go through the links in the document and analyze the videos. Fill out the remaining variables by grading the videos based on the headers at the top of the columns. Most of them are yes/no but some require a grade. Pretend you are an expert in the field looking at these videos for utility to patients

The columns that are yes/no are k, l, m, n, and o; p requires a grade as follows: "GQS

∗ 1 poor quality and flow, most information missing, and not useful for patients
∗ 2 generally poor quality and flow, with some information, and limited usefulness for patients
∗ 3 moderate quality and flow, with some important information sufficiently explained, and somewhat useful for patients
∗ 4 good quality and flow, most information present, and useful for patients
∗ 5 excellent quality and flow that is very useful for patients"

Analyze all linked videos regardless of the source; intended audience is medical professionals.”

A table with all of the video URLs and video characteristics was also provided, with the mDISCERN criteria listed for columns K through O.
